# Development of Hepatocellular Carcinoma Associated with Anabolic Androgenic Steroid Abuse in a Young Bodybuilder: A Case Report

**DOI:** 10.1155/2012/195607

**Published:** 2012-07-05

**Authors:** Aline Hardt, Dirk Stippel, Margarete Odenthal, Arnulf H. Hölscher, Hans-Peter Dienes, Uta Drebber

**Affiliations:** ^1^Department of Pathology, University Hospital of Cologne, Kerpener Straße 62, 50937 Cologne, Germany; ^2^Department of General Visceral and Cancer Surgery, University Hospital of Cologne, Kerpener Straße 62, 50924 Cologne, Germany

## Abstract

*Introduction*. Many different etiological factors are involved in the development of hepatocellular carcinoma (HCC). We report the case of HCC in a 37-year-old male professional bodybuilder with extensive anabolic androgenic (AAS) steroid abuse. *Case Presentation*. Because of increasing epigastric and abdominal pain, abdominal ultrasound was performed in a 37-year-old male professional bodybuilder. A hyperechoic lesion in the liver was detected in segment VI. The magnetic resonance imaging showed hepatomegaly and confirmed the lesion, which showed features of a hepatocellular adenoma (HCA). Laboratory values were inconspicuous. After laparoscopic segmentectomy the histological examination revealed HCC. *Conclusion*. While the development of HCA in the liver by chronic intake of AAS is well known, little is known about the association with HCC. The presented case may indicate aetiological association of chronic intake of AAS and the development of HCC.

## 1. Introduction

Hepatocellular carcinoma (HCC) is worldwide one of the most common malignant and widespread tumors with increasing incidence in the western world [1]. HCC represents the sixth leading cancer and the third most common cause of death from cancer [2]. 90% of primary malignant liver cell carcinomas are hepatocellular carcinomas [3]. Many different aetiological factors are involved in the development of HCC. Distribution of HCC varies considerably among geographic regions and ethnic groups. Males have higher liver cancer rates than females, ranging from 4 : 1 up to 8 : 1. In the majority of cases, HCC arises from chronic liver disease and cirrhosis (75–90%) [1]. Development of HCC is a multistep process, and major causes are the prevalence of chronic hepatitis B/C, chronic alcohol consumption, and aflatoxin exposure. Long-term use of oral contraceptives and high-dose anabolic androgenic steroids (AASs) are described in the literature as further, but rare, aetiologic factors [1, 3]. HCCs following AAS abuse are most often encountered in noncirrhotic livers [3]. Anabolic androgen steroids (AASs), synthetically produced hormones of the male testosterone, are often misused and self-administered by bodybuilders to rapidly increase muscle mass. The exposure leads to side effects in the cardiovascular, reproductive, musculoskeletal, and endocrine system [4, 5]. In the liver, 17*α*-alkylated AAS consumption induces cholestasis, peliosis hepatis, and liver tumors [5]. Different preparations are available for oral application with a quick effect and for injection for a long effective period [4]. Here, we present a case of a previously healthy 37-year-old male professional bodybuilder with HCC probably based on extensive anabolic abuse.

## 2. Case Presentation

**Figure 1:**
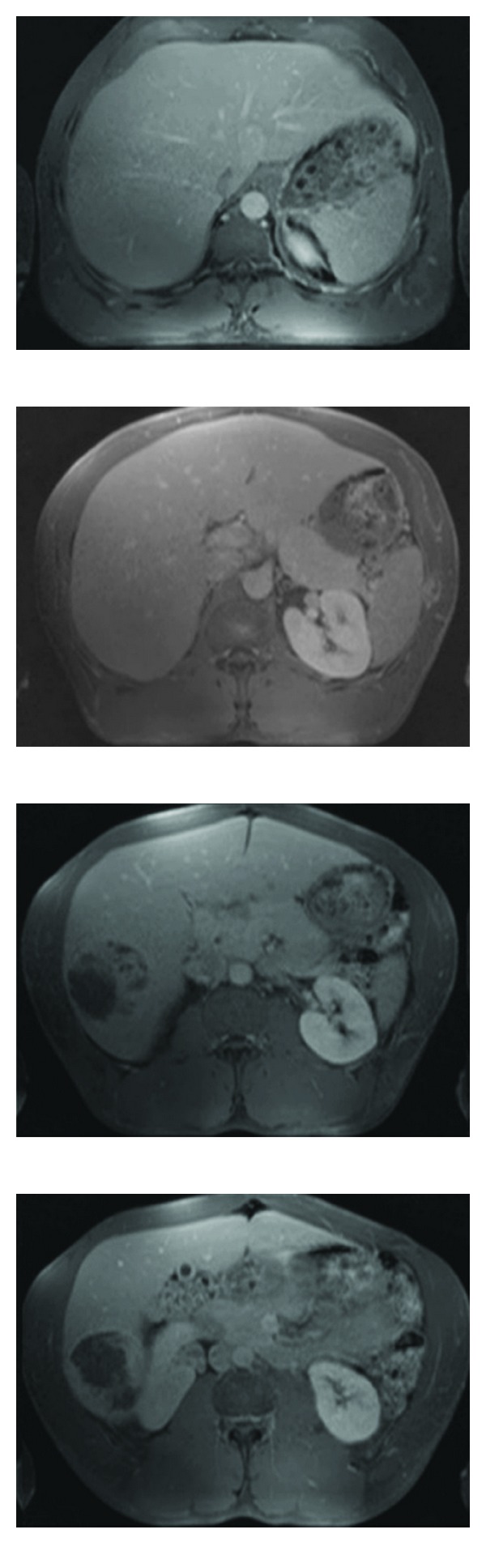
Magnetic resonance imaging showing the enlargement of the liver and the nonhomogeneous lesion in segment VI.

A 37-year-old male professional bodybuilder with a body height of 180 cm and a body weight of 118 kg presented himself with increasing epigastric and abdominal pain to an outside department of hepatology. The physical examination revealed an athletic appearance with enhanced muscular contour. A mild tenderness in the right upper abdominal quadrant was found. Any history of alcohol consumption or smoking was denied. Laboratory tests including liver function tests were normal. The serum alpha fetoprotein (AFP) level was 2 *μ*g/L (normal 5.8 *μ*g/L). A hepatic lesion was discovered by abdominal ultrasound. It appeared as a predominantly hyperechoic lesion in the right lobe of the liver (segment VI; 6 cm in maximal diameter). The magnetic resonance imaging showed a hepatomegaly and confirmed the lesion in segment VI (Figure 1), which showed features of a hepatocellular adenoma (HCA). The patient was transferred to the Department of Visceral Surgery, University of Cologne for a laparoscopic resection of the suspected HCA.

The patient reported AAS use with different kinds of anabolic substances and a stringent diet for increasing muscle mass. For a period of at least five years he has been consuming the following AASs in a daily medication schedule: Testosterone propionate, testosterone phenylpropionate, testosterone isocaproate, testosterone decanoate 250 mg, trenbolone acetate 75 mg, 5alpha-androstanediol 100 mg, boldenone and methandriol dipropionate 240 mg, 17*α*-Methyl-5*α*-androstano[3,2-c]pyrazole-17*β*-ol 100 mg, 17*β*-hydroxy-17*α*-methyl-2-oxa-5*α*-androstane-3-one 4 × 10 mg, letrozole 0,065 mg, and oxymetholon 3 × 50 mg or methandienone 10 mg. Oxymetholone or methandienone was discontinued three weeks before competition. In addition, he took spironolactone 100 mg, mesterolone 25 mg, fluoxymesterone 10 mg four weeks, and torasemide eighteen hours before competition. Furthermore, a daily intake of amino acid, vitamins, and mineral tablets, T4 (200 *μ*g), and growth hormones (8 I.E.) was reported. The nutritional protocol consisted of six small, protein-rich meals (chicken breast, fish, protein shakes, salad, vegetable, etc.), a high caloric and high protein-containing diet to build up muscle mass. Eight weeks before competition, the meals and consequently the caloric intake were reduced by 50% to reduce subcutaneous fat. Torasemide, a diuretic, was taken to achieve a more muscular bodily contour by reducing extracellular and subcutaneous tissue volume.

A laparoscopic segmentectomy of the liver was performed, and a 6 × 6 × 5 cm tumor was resected from the right hepatic lobe.

### 2.1. Pathological Findings

A partial hepatectomy specimen of 256 g, measuring 12.1 × 8.2 × 5.8 cm, was examined in the department of pathology. The surface was partly shiny, partly covered with white fibrous membranes with few adhesions. Slicing the specimen revealed a 6 × 6 × 5 cm tumor with central necrosis.

**Figure 2:**
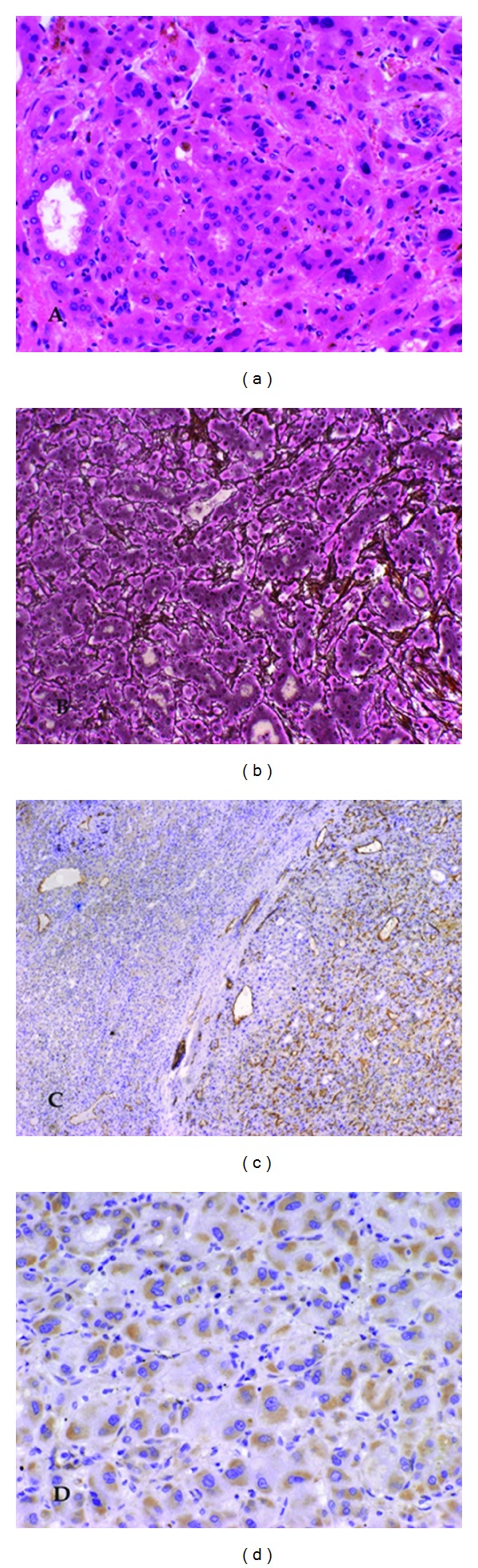
Histological features of hepatocellular carcinoma in a noncirrhotic liver. (a) Hepatocellular carcinoma with pseudoglandular pattern and bile production. (b) Loss of reticular fibres and cord thickening up to six cells. (c) Immunohistochemical staining for CD 34. (d) Positive immunohistochemical staining for glypican 3.

A histological examination was performed on formalin-fixed and paraffin-embedded sections: noncirrhotic liver parenchyma was infiltrated by an extensive necrotic carcinoma. The tumor consisted of trabeculae of medium-sized polygonal cells resembling hepatocytes separated by sinusoidal spaces and containing cytoplasmic bile inclusions. In places, pseudoglandular tumor cell formations were seen (Figure 2(a)). The trabeculae were surrounded by endothelial cells, which resembled capillary endothelium rather than normal hepatic sinusoidal endothelium. The cords varied in thickness consisting of up to six cells, and a loss of reticular fibres could be demonstrated by silver staining (Figure 2(b)). No capsule formation was seen; instead a pushing margin was found. Nuclear pleomorphism was moderate with cellular atypia, enlarged nucleoli, and a reduced amount of basophilic cytoplasm. The nuclei showed hyperchromatism and nuclear irregularity. The mitotic figure count was high with 2 to 6 mitoses per high power field (HPF = ×400). A vascular tumor invasion was not found. The surrounding liver parenchyma displayed minor signs of inflammation in the portal tracts but no cirrhosis.

### 2.2. Immunohistochemical Findings

Immunohistochemically, there was a diffuse and strongly positive cytoplasmic, granular staining in all tumor cells for CK 8 as well as for Hep-Par 1. CD 10 and CEA showed an intense canalicular staining pattern in the tumor cells. The endothelium, which surrounded the trabeculae, showed a clearly positive immunohistochemical staining for CD 34 (Figure 2(c)). A weaker, cytoplasmatic staining was seen for glypican 3 (Figure 2(d)). The tumor cells showed also a focal cytoplasmatic staining for CK 19. CK 7 was not expressed in the tumor cells. *β*-catenin, progesteron, and oestrogen receptors showed a weak nuclear staining. The mitotic index analysed by Ki-67 was low with 5-6% of positively stained cells.

A moderately differentiated HCC was diagnosed (TNM classification: pT1, pNX, pMX, L0, V0, R0).

### 2.3. Clinical Course

The clinical course was uneventful, and the patient was discharged on day 7. He has been followed up regularly by ultrasound and *α*-fetoprotein every three months (highest level 3 *μ*g/L) and magnetic resonance imaging every six months. He is doing fine after a follow-up period of 27 months without a sign of recurrence.

## 3. Discussion

We report the remarkable case of a young professional bodybuilder who has been consuming high doses of AAS over a long period of time and who developed hepatocellular carcinoma in the liver without AFP elevation.

Histopathological findings revealed unequivocal evidence of HCC with characteristic features like cytological atypia and a neoplastic trabecular pattern. As we have not been able to find HCA-like areas in the tumor, the mechanisms of carcinoma development remain elusive and have to be discussed.

It is well known that the liver is a hormone-sensitive organ expressing oestrogen and androgen receptors [3, 6]. HCAs as well as HCCs can arise in the context of synthetic steroid intake. The causative role for oral contraceptives in HCAs is well accepted, and approximately 320 new cases are diagnosed in Italy each year [2, 5, 7]. The development of de novo HCC as a consequence of anabolic steroid intake has been described in young patients with Fanconi's anemia who have been treated with anabolic steroids and who develop HCC at young age [8, 9]. In animal experiments a causative relationship between preneoplastic, benign, and also malignant lesions of the liver without attendant cirrhosis and treatment with anabolic-androgen steroids as well as with natural oestrogens could be demonstrated [3]. In this context Dombrowski et al. described an adenoma-carcinoma sequence [10]. In the present case the mechanism of carcinogenesis might be either “de novo carcinoma development” or “adenoma-carcinoma sequence.”

The adenoma-carcinoma sequence has recently been described for a subgroup of adenomas with *β*-catenin mutation [11]. HCA with *β*-catenin mutation reveals nuclear atypia and overexpresses *β*-catenin. In our case immunohistochemistry revealed *β*-catenin overexpression suggestive for carcinoma development from a preexisting HCA. However, *β*-catenin overexpression can be found also in “de novo HCC.” The patient has been AFP negative, which might be an argument for the adenoma-carcinoma sequence; however, there are AFP-negative de novo HCCs as well. Thus, the possibility of malignant transformation of an HCA cannot be proven in the case presented and we discuss that in our patient de novo carcinoma development is most probable.

A growing number of reports about abuse of anabolic androgen steroids in Western Europe and the USA by competitive and noncompetitive bodybuilders is reported in the literature especially since the 1990s [5]. A correlation between AAS use and HCA has been increasingly recognised in athletes taking AAS [5, 12, 13]. While danazol is associated with HCA formation, other preparations like oxymetholone, respectively, methyltestosterone can lead to HCC [8]. In addition to other synthetical androgenic steroids, our patient consumed the prospective carcinogenic oxymetholone (50 mg) three times a day.

In the literature the correlation between anabolic misuse by bodybuilders and the development of HCC is reported only in seven cases retrospectively. In contrast, numerous cases about the development of HCC in patients taking anabolic steroids because of other underlying diseases have been referred [8, 9].

Bodybuilders, who abuse anabolic androgen steroids over a long period of time have a great risk of developing an HCA or HCC and should therefore be well monitored. Periodic hepatic ultrasound seems to be an adequate screening procedure to detect the development of hepatic lesions [13]. Although most of the tumors developing by AAS misuse or intake of oral contraceptives are benign, early detection is important in order to avoid the associated risk of malignant transformation and life-threatening haemorrhages [14]. In these cases a surgical excision is recommended [5, 13]. A nonsurgical approach for these tumors has also been suggested because of the regression of some tumors after stopping the medication. In contrast to this observation, a recurrence and enlargement have been reported in cases where steroids have been continued [9, 15].

## 4. Conclusion

In conclusion, this case demonstrates the development of HCC in a noncirrhotic liver probably based on the chronic intake of AAS. The mechanisms of carcinoma development in this case remain elusive. Bodybuilders should be aware of the risk of carcinoma development when abusing AAS.

##  Consent

Written informed consent was obtained from the patient for publication of this case report and accompanying images. A copy of the written consent is available for review by the Editor-in-Chief of this journal.
